# Impact of a 5-Day Expedition to Machu Picchu on Persons with Multiple Sclerosis

**DOI:** 10.1155/2014/761210

**Published:** 2014-05-21

**Authors:** Marie Beatrice D'hooghe, Peter Feys, Sam Deltour, Isabelle Van de Putte, Jan De Meue, Daphne Kos, Bert O Eijnde, Paul Van Asch

**Affiliations:** ^1^Department of Neurology, National MS Center, Vanheylenstraat 16, 1820 Melsbroek, Belgium; ^2^Faculty of Medicine and Pharmacy, Vrije Universiteit Brussel, Pleinlaan 2, 1050 Brussel, Belgium; ^3^Rehabilitation Research Center (REVAL), Biomedical Research Institute, Faculty of Medicine and Life Sciences, Hasselt University, Agoralaan Building A, 3590 Diepenbeek, Belgium; ^4^Faculty of Medicine, University Leuven, Oude Markt 13, 3000 Leuven, Belgium; ^5^Department of Physiotherapy, National MS Center, Vanheylenstraat 16, 1820 Melsbroek, Belgium; ^6^General Medicine, Den Blink, J.B. Nowélei 19, 1800 Vilvoorde, Belgium; ^7^Occupational Therapy, Plantijn Antwerp & KU Leuven, University of Leuven, Oude Markt 13, 3000 Leuven, Belgium; ^8^Fit Up (Physiotherapy Center), Mechelsesteenweg 192/A, 2550 Kontich, Belgium

## Abstract

Persons with multiple sclerosis (MS) are less physically active than nondiseased persons and often report low self-efficacy levels. In the context of an awareness project to promote physical activity and participation in MS, we addressed the impact of training for and participation in a unique expedition. Medical events, relapses, and self-reported neurological worsening were followed from 6 months before and up to 4 months afterwards. Validated patient-reported outcome measures were used to assess fatigue, self-efficacy in exercising, walking abilities, and illness perception. Nine participants completed the training, expedition, and observational study. Minor events, relapses, and/or neurological worsening were reported in six participants. The three participants with mild disability and no cardiovascular risk factors or comorbidities were free of medical and neurological events. We found a significant reduction of motor fatigue at last when compared with the first assessment. The reduction tended to be more evident in participants with mild disability (Expanded Disability Status Scale (EDSS) <4 at baseline). Cognitive fatigue, self-efficacy, and self-reported walking abilities did not change significantly. Illness perceptions tended to be reduced over time in the domains of consequences, identity, and concerns. Overall, no major adverse events occurred.

## 1. Introduction

The benefits of physical activity for overall health have been extensively documented. Persons with multiple sclerosis (MS), even when having limited neurological disability, appear to be less physically active when compared to age-matched controls [1].

Therefore, they may lack, at least partially, the positive effects of physical activity on general health and MS-related changes in body structure, body functions, activities, and participation [2]. The beneficial effect of exercise training on walking mobility among individuals with MS has been extensively documented [3] and the evidence of improvements in quality of life and fatigue is supportive [2, 4]. Although the underlying mechanisms remain to be elucidated, vascular comorbidities have been associated with an increased risk of disability progression in MS [5]. Taken together, increasing physical activity levels in MS may be important to reduce morbidity and disability progression.

Unfortunately, the barriers to be physically active are multiple and include illness perceptions that exercise might facilitate MS relapses [6]. Meanwhile, it has been shown that intensive physical exercise does not lead to increased relapse rate [7]. Other barriers relate to the perception that exercise is fatiguing [6]. Fatigue, one of the most prevalent MS symptoms, occurs in up to 80% of persons with MS and is the most disabling symptom in many of them. No consensus on the relationship between fatigue and disease-related variables has been reached [8]. A link with depression and anxiety has been suggested [9]. Motivational aspects might also be implicated [10] and reward responsiveness [11] has been suggested. Reduced self-efficacy for exercise and lack of appropriate infrastructure were equally perceived as barriers by persons with MS living in the community [6]. In view of the multiplicity of barriers, professional guidance has been proposed to motivate and support persons with MS to be physically active [12].

In the context of an awareness project to promote physical activity and participation in MS, PvA initialized an Andes trekking for 10 persons with MS, with Machu Picchu (Peru) as final destination, http://www.msmachupicchu2012.be/. The opportunity of providing professional coaching and medical support during training, travelling, and expedition was taken to prospectively collect data on disease activity, fatigue, illness perception, walking abilities, and self-efficacy for exercise during a 10-month period.

## 2. Methods

### 2.1. Participants

Ten candidates were recruited by neurologists and physiotherapists affiliated with the MS Center in Melsbroek and the fitness center “Fit up” in Kontich, Belgium. Only patients with a mild or moderate degree of neurological disability due to MS and a high potential of motivation were considered. After the first meeting in March 2012, one woman decided not to continue. Among other reasons, she did not want to have her diagnosis to be known in the public domain.

Nine accompanying persons with a medical or physiotherapeutic background were also involved from the start: 3 physiotherapists, 1 exercise physiologist, 2 coaches, 1 general physician, 1 neurologist, and 1 medical student.

### 2.2. Study Protocol

The study protocol was approved by the leading ethical committee of the University Hospital Leuven and the local ethical committee of the MS Center, Melsbroek, Belgium.

**Figure 1:**
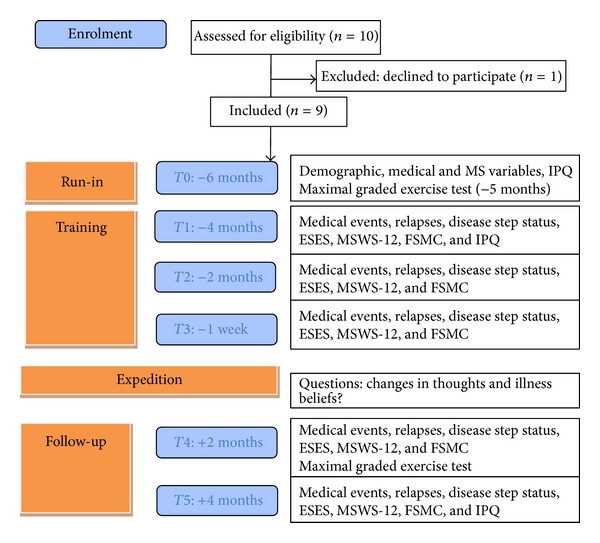
Enrolment, timeline, and assessments at different time points.

Longitudinal data were collected on medical and MS variables, fatigue, self-efficacy, walking disability, and illness perception. An overview of the timeline and assessments is given in Figure 1.

At the first meeting, defined as time point 0 (*T*0, 6 months prior to the expedition) demographic, medical, and MS variables were collected, including the Expanded Disability Status Scale (EDSS) [13], assessed by the treating neurologist. Self-reported medical events, relapses and disability, fatigue, walking ability, and self-efficacy for exercise were registered every 2 months resulting in 5 assessments: 1 at the start of the training (*T*1), 2 during the training period (*T*2, *T*3), and 2 following the expedition (*T*4, *T*5). Illness perceptions were assessed at 3 time points (*T*0, *T*1, and *T*5). Nine participants signed the informed consent.

### 2.3. Outcome Measures

We used the self-assessment scale of disability developed for the European study on costs and quality of life in MS [14, 15], which is based on the original validated description in the Expanded Disability Status Scale (EDSS) [13] and on the Patient Determined Disease Steps instrument (PDDS) [16]. Subjects are asked to select from a series of statements describing limitations and walking disability. The self-assessment scale of disability allows participants to be categorized into 11 steps of disability, from 0 to 10, corresponding to EDDS scores [15].

The “Fatigue Scale Motor Cognition” (FSMC) [17] was used to assess MS-related cognitive and motor fatigue in MS in 20 items. This highly sensitive and specific scale has adequate reliability and validity. Cut-off values for the composite score and the cognitive and motor components allow patients to be classified as mildly, moderately, or severely fatigued.

The “MS walking ability scale” (MSWS 12) [18, 19], a reliable and valid patient-reported measure, assesses the impact of MS on walking ability. The “Exercise Self–Efficacy Scale” (ESES) [20] is a 10-item questionnaire that uses a 4-point Likert's scoring system. Higher scores are indicative of higher perceived exercise self-efficacy. The ESES is reported to be reliable and valid in patients with spinal cord injury [20] and has been used previously in MS patients [6, 21].

The brief version of the Illness Perception Questionnaire (IPQ) [22] assesses the beliefs that individuals construct about their disease. Five items assess cognitive illness representations, 2 items assess emotional representations, and 1 item assesses illness comprehensibility. A sum score can be calculated. The scale has a good test-retest reliability and concurrent validity with relevant measures [22].

To improve our understanding of how participants experienced this expedition, a questionnaire was developed. Potential changes in thoughts, illness beliefs, and self-esteem were asked, as well as the contributing factors and messages for the MS community.

Results were analyzed descriptively given the small sample size. For some analyses, a distinction was made between persons with EDSS 4 at baseline compared to those with a lower score. Data were also analyzed using a nonparametric test. We used the Wilcoxon signed-rank test because measurements of continuous dependent variables were available. We compared between different time points before and after the intervention for the group.

### 2.4. Exercise Test and Training Period

Five months prior to the expedition, subjects performed a maximal graded exercise test on an electronically braked cycle ergometer (eBike Basic, General Electric GmbH, Bitz, Germany). Ventilatory parameters and heart rate were continuously monitored by a 12-lead ECG device (Jaeger Oxycon, Erich Jaeger GmbH, Germany). Following a 10 min warm-up at 40 W, subjects were seated on cycle ergometer for three minutes to obtain resting data. Next, subjects were instructed to cycle at a rate of minimum 70 rpm, against a starting workload of 40 W. Hereafter, workloads were increased every 3 min by 40 W (♂) and 30 W (♀) until voluntary exhaustion. Just before workloads were increased, heart rate was registered, and capillary blood lactate (Accutrend Plus, Roche Diagnostics Limited, Sussex, UK), oxygen uptake, and respiratory exchange ratio were measured.

Based on the results, a personalized training schedule was provided in May 2012 (*T*1). Group meetings were planned to evaluate walking capacities and training progression of the participants.

The maximal graded exercise test A was repeated 6 weeks after the expedition.

### 2.5. Salkantay Expedition

On September 20, 2012, the group flew from Brussels, Belgium, to Lima, Peru. A flight to Arequipa (2,300 m) and a trip to the Colca Canyon valley, getting over 4,900 m, introduced altitude experience. The flight to Cusco (3,400 m) and a bus trip on September 27 brought the group to the start of the 5-day Salkantay trekking. The estimated altitude at start, altitude meters, walking time, and distance are given in Table 1 (data obtained by Garmin GPS).

Walking was also measured by accelerometer (Actibelt, Sylvia Lawry Center, Munich, Germany). The performance of participants was monitored by heart rate measurements (POLAR) and regular self-reported rating of walking ability and energy levels, to adapt walking pace and rest breaks when necessary. Horse assistance was provided when necessary and feasible (steepness) during the first 3 days. Because the effort was estimated to be too strenuous, parts of the trail during the last 3 days have been done by bus.

## 3. Results

### 3.1. Participants

Baseline characteristics of the nine participants with relapsing MS are found in Table 2. The three participants with EDSS 4 presented with cardiovascular risk factors (two with smoking and one with obesity).

Training schedules started from low levels in 3 (group 3), from intermediate levels in 4 (group 2), and from high levels in 2 participants (group 1). Each group contained one male representative.

### 3.2. Clinical Observations

During run-in (*T*0-*T*1), one subject was treated with steroids for a relapse. Two other subjects developed a relapse during the training phase (*T*1–*T*3). One of them was treated with pulse steroids. Another subject was treated with antibiotics because of a bronchial infection.

During travelling, one patient developed gastroenteritis in Arequipa (Peru). Another patient experienced increased fatigue and dizziness after having crossed high altitudes (>4000 m). Most subjects got some experience with altitude sickness at altitudes of 3600 m or more. Treatment with analgesics was given if needed. During the 5-day trekking, transient sensory complaints were mentioned and increased equilibrium disturbances were observed in several participants, without evidence for a relapse. Walking measurement by accelerometer revealed, across 5 days, an average step count of 17409 with an average daily step ratio of 94, 8% between start and end.

In the months following the expedition, four patients reported increased neurological symptoms, suggestive of a relapse. For two subjects, this was the second episode. One of them was treated with pulse steroids at the two occasions.

### 3.3. Exercise Capacity

Compared to PRE, heart rate at 40 W (PRE: 117 ± 14 b/m versus POST: 108 ± 12 b/m, *P* = 0.09) and at the aerobic threshold (PRE: 137 ± 8 b/m versus POST: 141 ± 9 b/m, *P* = 0.08) tended to be, respectively, lower and higher following the training and expedition period. Furthermore, POST workloads at the anaerobic threshold (PRE: 123 ± 43 W versus POST: 132 ± 41 W, *P* < 0.05) were lower and peak blood lactate concentrations were higher after the training and expedition period (PRE: 8.44 ± 1.0 mmol/L versus POST: 9.6 ± 1.3 mmol/L, *P* < 0.05).

### 3.4. Outcome Measures

#### 3.4.1. Self-Assessment Scale of Disability

Although fluctuations in self-reported disease steps were observed in all subjects, only the three participants with EDSS 4 at baseline reported a sustained increase. In two of them, this increase followed a very low, self-assessed disease step at baseline (0 or 1). No further worsening was reported after *T*3.

#### 3.4.2. Fatigue

Overall, median values of the FSMC sum transiently declined (Table 3).

When using the Wilcoxon signed-rank test to compare fatigue scores at the different time points, a statistically significant difference was obtained for motor fatigue when comparing *T*1 and *T*5 (*P* = 0.007). Median values for motor fatigue appeared to be reduced persistently in participants with mild disability (EDSS < 4).

**Figure 2:**
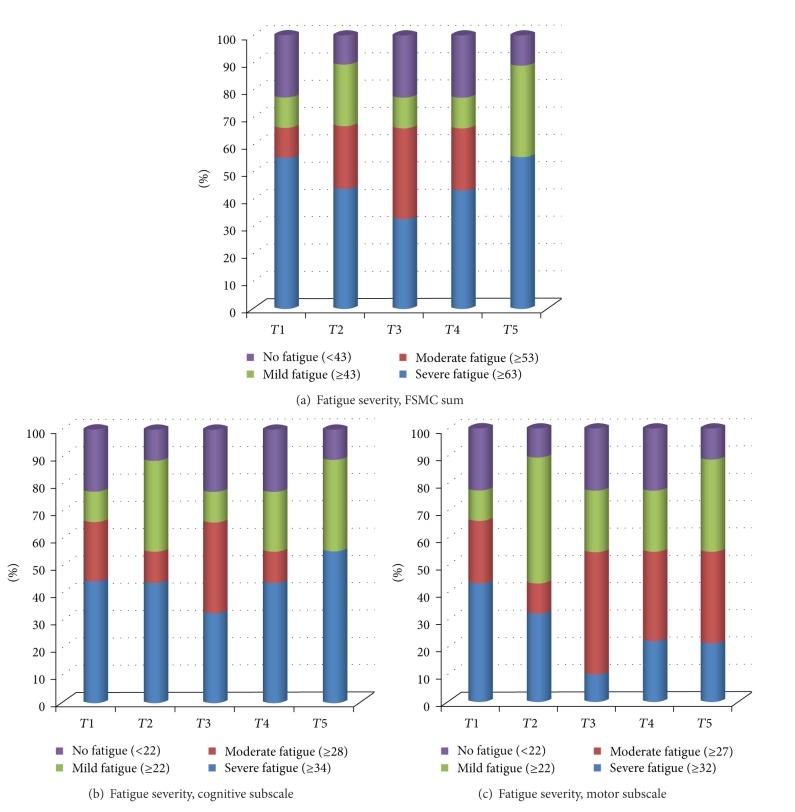
Longitudinal data of the number of patients with their graded fatigue for the sum, cognitive and motor subscales of the FSMC. The timeline of the 5 assessments is presented in the X axis. The percentage of participants is presented in the Y-axis. Different colours are indicating the severity, based on cut-off values, as indicated. The number of severely fatigued participants changed over time, especially for the motor subscale. Four subjects started with severe motor fatigue, including 2 subjects with EDSS 4 (*T*1). A transient reduction occurred in 3 of them, just before travelling to Peru (*T*3). Severe motor fatigue came back in 1 subject, which results in 2 subjects with severe motor fatigue (*T*5), both with EDSS 4 at baseline.

Longitudinal data of the number of patients with their graded fatigue for the sum, cognitive, and motor subscale of the FSMC are shown in Figure 2. When compared with baseline, fewer participants reported severe MS-related fatigue just before the expedition (*T*3).

#### 3.4.3. Walking Ability and Self-Efficacy for Exercise

Overall, self-reported walking abilities and self-efficacy for exercise did not change significantly over time. However, the higher median values of the MSWS 12 in participants with EDSS 4 at baseline tended to decline transiently.

#### 3.4.4. Cognitive Illness Representations

**Figure 3:**
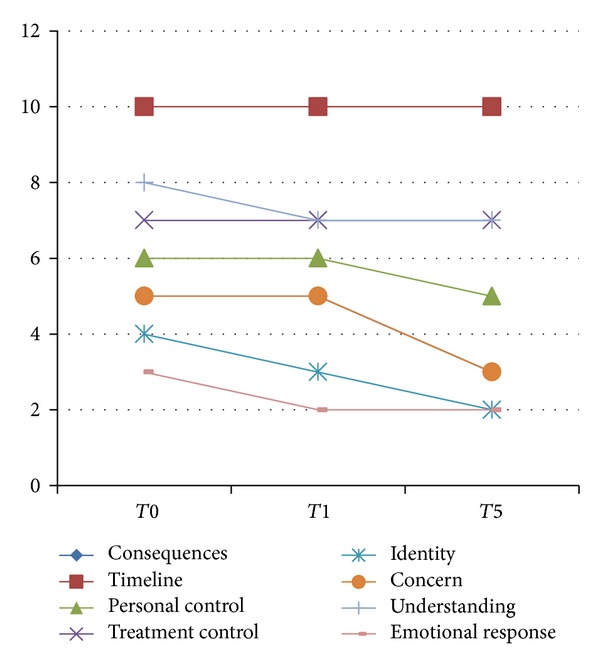
Graphical representation of longitudinal data on IPQ items. The timeline of the 3 assessments is presented in the *X*-axis. The median values for each item are presented in the *Y*-axis. Different colours are indicating the items. Item 1 (consequences) coincides with item 6 (concern). A reduction of 2 points is observed for the items 1 and 5 (consequences and identity), assessing cognitive illness representations. The item “illness identity” is a measure of the signs and symptoms perceived by the person to be part of the disease. The item “illness consequences” refers to the perceived effects and outcomes of illness. The median values of this item coincide with those of item 6 (concern). The item “concern” (item 6) assesses emotional representations.

Median values with interquartile ranges are presented in Table 4. A graphical presentation of the median values is shown in Figure 3. There were no significant differences when using the Wilcoxon signed-rank test.

#### 3.4.5. Questionnaire

Participants described this expedition as heavy, both physically as mentally. Motivation and training were thought to be essential. Several subjects reported a less dominant perception of MS as they expressed having a life with MS, to make their own decisions and get respect from family and friends. Although most participants reported feeling stronger, some of them also expressed the hard confrontation with limitations due to MS. The experience of wellbeing when being physically active, the support from family, friends, and MS community, and professional coaching were experienced as very helpful and encouraging. The messages to other people with MS included advices to be physically active, to be open for new experiences, and to never give up.

## 4. Discussion

In the context of an awareness project to promote physical activity and participation in MS, we obtained prospective information on medical and disease variables, fatigue, illness perception, walking abilities, and self-efficacy for exercise from 6 months before and up to 4 months after the expedition in the 9 selected candidates. Three minor medical events (3 subjects), 7 relapses (5 subjects), and sustained neurological worsening (3 subjects) were reported in six participants. We found a significant reduction of motor fatigue at the last assessment (*T*5) when compared with the first (*T*1). When stratifying the results based on the degree of disability (having reached EDSS 4 or not), the persistence of motor fatigue tended to be more evident in participants with mild disability. Cognitive fatigue, self-efficacy, and self-reported walking abilities did not change significantly. The perceived illness consequences, identity, and concerns appeared to be declined over time.

The relapse rate during the observational study period did not markedly differ from the relapse rate in the year before. Three of the five participants treated with immunomodulatory drugs developed relapses. Steroids were given for 2 of the 3 MS relapses before and for 1 of the 4 relapses after the expedition. The three subjects with self-reported neurological worsening at *T*3 had reached EDSS 4 at baseline. Because they reported no further worsening at later time points, their estimation at *T*3 may have been more realistic than initially. Remarkably, the three participants, with no cardiovascular risk factors or comorbidities and mild disability at baseline, were event-free.

Although we have not measured physical activity in the community, the tentative changes in exercise tests before and after the expedition support a training effect. During the trekking, the efforts by participants, as based on heart rate monitoring and step counts, corresponded to highly active walking when compared to habitual community walking [23].

The observation of reduced fatigue in association with training is in line with previous findings [2]. A recent systematic review of interventional studies concluded that physical exercise has the potential to reduce fatigue in MS [4]. However, findings were heterogeneous and only a few studies have evaluated fatigue as the primary outcome measure. The FSMC scale is a new scale [17] and is able to consistently distinguish between motor and cognitive components of fatigue in MS. Even though both fatigue components are not completely independent, increased physical activity for several months is a strong candidate to reduce motor fatigue. However, longitudinal data are limited. A treatment effect of natalizumab has been reported over 12 months, with parallel declining scores for motor and cognitive fatigue components [24].

Our findings suggest differences in the pattern of changes in motor versus cognitive fatigue in relation to training and disability. The reduction of motor fatigue appeared to be more evident in mildly disabled patients whereas the severity of cognitive fatigue remained rather stable over time in all participants. This has not been reported previously. Because of the correlations of the motor fatigue subscale with EDSS [10] and the start of the progressive phase of MS around EDSS 4, changes in subjects with moderate disability may be more difficult to obtain and to maintain.

The role of motivation, participation, professional coaching, social support, illness beliefs, reduced anxiety and depression, or a combination of these factors may also have played a role in reducing fatigue before and/or increasing fatigue after the expedition. As stated in the systematic review, the social interaction component, present in many positive studies, may affect the perception of fatigue [4]. Participants in our study not only increased their levels of physical activity but also played an ambassador role in informing the MS community.

Overall, there were no longitudinal changes in the MSWS-12 and the SES for exercise. However, in the subgroup of subjects with higher disability, the MSWS-12 scores transiently tended to decline before returning to baseline levels at the final assessment. The validation of the MSWS-12 in MS patients with, on average, a substantial mobility limitation suggests that the sensitivity to detect changes in mild MS may be limited [18, 25]. Instead of changes in perceived walking ability, they may have changed overall physical activity levels reflecting habitual performance. The overall high scores on the SES were indicative of a high self-efficacy for exercise. This probably reflects coping behaviour with regard to exercise. The belief in one's capabilities to take the necessary actions required for adhering to exercise has been strongly associated with physical activity in a recent longitudinal study of individuals with MS [26]. The significance of the final decline in self-efficacy in subjects with moderate disability remains to be determined.

The assessments of cognitive and emotional representations of illness suggest a reduction in the perceived adverse consequences, the signs and symptoms perceived to be part of MS (identity), and the expression of concerns. Together with the message for the MS community to be open for new experiences, expressed by several participants, our data suggest a less dominant and less negative perception of MS.

When diagnosed with MS, uncertainty and potential functional loss are leading to overall anxiety and threatening health-related quality of life. In an attempt to make sense of this illness experience, individuals construct cognitive models about MS. These beliefs are thought to be important for determining an individual's response [27], which is then appraised for efficacy. Evidence suggests that illness representations relate to later outcomes in several conditions [28–30]. While cross-sectional studies have reported associations with health-related quality of life, adjustment, depression, self-esteem, and activities of daily life [27, 30, 31], no longitudinal data have been published in MS. Interventions addressing negative illness cognitions may potentially improve health-related outcomes. In the context of our study, participation and professional guidance of persons with MS including information, motivation, and support may have contributed to changing these beliefs.

We fully acknowledge the limitations of this exploratory study. First, the number of participants is limited. Second, individuals were selected on motivation and disability, given that this was the first expedition that focused on feasibility rather than proposing a standard intervention for all. We cannot generalize our findings to persons with MS with severe disability, even though we aim to encourage all persons with MS to be physically active and to participate in the community with an active lifestyle. Third, the intervention included but was not limited to physical training. In the absence of a control group, we cannot exclude random or time-dependent changes in the severity of MS-related fatigue or illness perceptions. Fourth, the small sample did not allow us to look for interaction effects in participants with mild and moderate disability (*n* = 2 only). Fifth, the occurrence of medical events, relapses, and neurological worsening was primarily based on judgement by the participant. Nevertheless, confirmation by the treating physician was asked and information about MS diagnosis, comorbidities, and EDSS was obtained from the treating neurologist.

## 5. Conclusions

This 10-month observational study allows concluding that physical training and participation to a unique expedition has been feasible for the selected individuals with MS. Overall, minor events were observed in two thirds of the participants. All of them either had moderate disability, cardiovascular risk factors, and/or comorbidities at baseline. Motor fatigue was significantly reduced. This effect tended to be more evident in participants with mild disability.

Illness beliefs tended to be reduced including a less dominant perception of MS.

Further studies in MS are needed to address the role of disability, disease stage, cardiovascular risk factors, and comorbidities in training and the severity of fatigue. Distinguishing motor and cognitive fatigue and assessing illness perceptions may help to clarify the underlying mechanisms of MS-related fatigue.

## Figures and Tables

**Table 1:** Description of the Salkantay expedition.

	Starting point	Reference during walking	Altitude at start	Walking time	Distance (km)
Day 1	Mollepata	West of Rio Blanco	3348 m	>6 hours	13.0
Day 2	Soraypampa	Across the Salkantay pass, 4600 m	3920 m	>8 hours	16.0
Day 3	Inca campsite Huayramachay	Across Rio Santa Teresa	3288 m	3 hours	4.4
Day 4	Hydroelectrica Train station	Rio Urubamba	1880 m	4 hours	9.0
Day 5	Aguas Calientes	Machu Picchu	1960 m	3 hours	3.1

**Table 2:** Baseline characteristics of the participants (*n* = 9).

Female number (%)	6 (66%)
Relapsing onset (%)	9 (100%)
Age at trekking, year	
Median (range)	42 (28–49)
Age at onset of MS, year	
Median (range)	30 (23–40)
Disease duration, year	
Median (range)	9 (3–24)
EDSS	
Median (range)	3 (1–4)
Annualized relapse rate	0.80
Current treatment	
Interferon beta or glatiramer acetate	3 (2M 1F)
Natalizumab or alemtuzumab	2 (2F)
No immunomodulatory treatment	4 (1M 3F)
Comorbidity (rheumatoid arthritis, narcolepsy)	2 (2F)
Cardiovascular risk factors (smoking, obesity)*	4/9 (2M, 2F)

*Three of them had reached EDSS 4.

**Table 3:** FSMC, ESES, MSWS 12, and brief IPQ, median values (range) and stratified by EDSS 4.

	*T1 *	*T*2	*T*3	*T*4	*T*5
FSMC sum					
ALL (*n* = 9)	68 (23–79)	59 (29–79)	61 (24–79)	59 (27–82)	69 (26–84)
EDSS < 4 (*n* = 6)	68 (23–79)	64 (29–79)	61 (24–79)	62 (27–77)	61 (26–77)
EDSS = 4 (*n* = 3)	61 (34–68)	59 (48–72)	58 (38–76)	59 (40–82)	69 (48–84)
FSMC motor					
ALL (*n* = 9)	31 (10–36)	26 (10–37)	28 (10–37)	28 (13–40)	28 (10–40)
EDSS < 4 (*n* = 6)	30.5 (10–36)	26 (10–33)	25.5 (10–30)	26.5 (13–30)	26 (10–30)
EDSS = 4 (*n* = 3)	32 (14–33)	32 (25–37)	29 (18–37)	33 (18–40)	35 (22–40)
FSMC cognitive					
ALL (*n* = 9)	32 (12–47)	33 (19–47)	31 (14–49)	29 (14–47)	34 (16–48)
EDSS < 4 (*n* = 6)	35 (12–47)	36 (19–47)	32 (14–49)	34 (14–47)	35 (16–48)
EDSS = 4 (*n* = 3)	29 (20–35)	27 (27–35)	29 (20–39)	26 (22–42)	34 (26–44)
MSWS					
ALL (*n* = 9)	14 (12–39)	13 (12–33)	13 (12–26)	13 (12–31)	14 (12–38)
EDSS < 4 (*n* = 6)	13 (12–18)	12.5 (12–15)	12.5 (12–15)	12.5 (12–15)	12 (12–15)
EDSS = 4 (*n* = 3)	30 (18–30)	21 (16–33)	23 (13–26)	25 (16–31)	31 (17–38)
ESES					
ALL (*n* = 9)	36 (27–40)	37 (34–40)	37 (33–40)	37 (33–40)	37 (28–39)
EDSS < 4 (*n* = 6)	36 (32–40)	36.5 (34–39)	37 (34–40)	37 (35–39)	37 (35–38)
EDSS = 4 (*n* = 3)	37 (27–40)	37 (36–40)	35 (33–40)	35 (33–40)	29 (28–39)

**Table 4:** Brief IPQ median values (interquartile range).

	*T*0	*T*1	*T*5
Overall	49 (40–51)	43 (34–50)	43 (35–51)
Consequences	5 (3–7)	5 (3–7)	3 (3–5)
Timeline	10 (10-10)	10 (10-10)	10 (10-10)
Personal control	6 (4–7)	6 (5-6)	5 (4–7)
Treatment control	7 (6–8)	7 (6–8)	7 (6–8)
Identity	4 (3–5)	3 (2–5)	2 (2–4)
Concern	5 (3–6)	5 (4-5)	3 (2–6)
Understanding	8 (7–9)	7 (6–9)	7 (7-8)
Emotional response	3 (2–6)	2 (2–4)	2 (1–8)

## Abbreviations

MS: Multiple sclerosis
EDSS: Expanded Disability Status Scale
*T*: Time point
FSMC: Fatigue Scale Motor Cognition
MSWS: MS walking ability scale
ESES: Exercise Self-Efficacy Scale
IPQ: Illness Perception Questionnaire.
